# Night Shift and Decreased Brain Activity of ICU Nurses: A Near-Infrared Spectroscopy Study

**DOI:** 10.3390/ijerph182211930

**Published:** 2021-11-13

**Authors:** Noelia Durán-Gómez, Jorge Guerrero-Martín, Demetrio Pérez-Civantos, Casimiro Fermín López-Jurado, Jesús Montanero-Fernández, Macarena C. Cáceres

**Affiliations:** 1 Departamento de Enfermería, Facultad de Medicina y Ciencias de la Salud, Universidad de Extremadura, 06006 Badajoz, Spain; jorguerr@unex.es (J.G.-M.); casimirolj@unex.es (C.F.L.-J.); mcaceres@unex.es (M.C.C.); 2 Facultad de Medicina y Ciencias de la Salud, Universidad de Extremadura, Hospital Universitario de Badajoz, 06006 Badajoz, Spain; dperciv@unex.es; 3 Departamento de Matemáticas, Facultad de Medicina y Ciencias de la Salud, Universidad de Extremadura, 06006 Badajoz, Spain; jmf@unex.es

**Keywords:** nurses, night, shift, sleep, anxiety, dorsolateral prefrontal cortex, near-infrared spectroscopy, cerebral blood flow, cognitive, performance

## Abstract

Background: Shift working is associated with a profound desynchronization of circadian rhythm and in particular, night-shift work disrupts normal circadian physiology. Sleep deprivation affects the functioning of certain brain areas and thus impairs cognitive performance. The purpose of this study was to investigate the effects of the night shift on cognitive performance and cerebral oxygenation/haemodynamics. Methods: A prospective, observational, comparative, randomized and cross-over study was carried out. A total of 74 intensive care unit nurses in Spain were included in the study. The following variables were measured: sociodemographic, burnout, anxiety, baseline cerebral oxygenation levels on night and day shift using a near-infrared spectroscopy system and cognitive task performance during a verbal fluency task to evaluate the alterations in the prefrontal cortex, assessed as changes in regional saturation index. Results: The average regional saturation index decreased significantly in the night shift (r = 0.560, *p* < 0.001). The ICU nurses showed a significant decrease in the verbal fluency test on average (8.53 ± 8.49, *p* < 0.001) and, in general, there was also a significant increase in anxiety score (3.17 ± 7.56, *p* = 0.001). Conclusions: Sleep deprivation during the night shift was considered to be related to decreased dorsolateral PFC reactivity. After the night shift, the nurses showed a decrease in prefrontal cortex activity and in cognitive performance.

## 1. Introduction

Nursing care provision is always organized in shift work to guarantee the healthcare of patients. Sleep deprivation and chronic tiredness related to long working hours are common among health services, mainly intensive care unit (ICU) nurses. This problem is increased by night-shift-related acute fatigue and sleep deprivation. It has been clearly established that chronic tiredness, fatigue and sleep deprivation have negative impacts on the health status of nurses and can jeopardize patient safety [1,2]. In fact, the main reasons for medical errors are stress, fatigue, increased workload, night shifts, and workflow interruptions.

Shift working is associated with a profound desynchronization of circadian rhythm [3] and in particular, night-shift work disrupts normal circadian physiology [4]. When people engage in rotating or night shift work, the circadian rhythms are unable to quickly adapt to a rapidly changing activity schedule. Sleep duration may be even less between consecutive night shifts, because the internal biological (circadian) clock reduces sleep drive during the daytime. This is of concern because of the impact of reduced sleep duration on cognitive function in nurses, especially when superimposed on the circadian dysfunction inherent in night-shift work [5]. Sleep deprivation and misalignment of the circadian phase are also linked to slow reaction time, frequent attention lapses and increased error rates in carrying out tasks.

At the same time, the misalignment of circadian rhythms and, subsequently, sleep deprivation or disorders can promote the onset of burnout. This burnout can also be associated with shift work carried out for many years, and with the fact that sleeping a few hours during the day coupled with spending less than eight hours away from work can produce negative effects, especially in emotional domains [6,7]. Based on possible causes, burnout is classified into three types: (1) personal burnout (CBIpb), which is associated with subjective and/or personal problems, such as sleep disorders; (2) work-related burnout (CBIwb), which is associated with factors of a poor work climate; and (3) patient-related burnout (CBIcb), which is associated with emotional involvement in a patient’s problems [8]. Shift work places nurses under stress and can cause their health, well-being and lifestyle to deteriorate. Nurses who feel strained are more likely to experience anxiety, depression, somatic disturbances, sleep disorders and burnout [9]. In fact, recent research [10] shows that providing continuity of care versus non-continuity models (rotating shifts) is related to lower levels of burnout and anxiety.

Furthermore, sleep deprivation affects the functioning of certain brain areas and thus impairs cognitive performance. Several hypotheses have been proposed to explain the reasons for decreased cognitive performance associated with sleep deprivation due to prolonged wakefulness. Cognitive impairments may be mediated predominantly through the decreased alertness and attention that occurs with wake-state instability [11,12]. Impairments may occur in several domains, including attention, cognition, motor skills, and mood [13]. Some authors have suggested that sleep deprivation particularly impairs the cognitive performances that are associated with the prefrontal cortex (PFC), including higher functions, and a number of neuroimaging studies focusing on sleep deprivation have revealed the inappropriate activation of predominantly the prefrontal regions in the working memory system [14,15,16,17]. Functional brain imaging studies have revealed that sleep deprivation is characterized by reductions in brain metabolism, with the greatest reductions evident in PFC, understood as the deactivation of the PFC [18], perhaps referring to a problem with the self-regulation of the PFC in healthy individuals. Specifically, sleep deprivation has been reported to impair performance in cognitive tasks, including verbal fluency tasks (VFT), that are putatively dependent upon PFC involvement, and that some cerebral systems, particularly PFC, would be less activated by cognitive tasks in sleep-deprived (night shift) than in rested subjects (day shift).

Near-infrared spectroscopy (NIRS), a non-invasive functional neuroimaging technology widely used in recent years, can measure haemodynamic changes over the surface of the cortices of the bilateral fronto-temporal regions [19,20]. This technique permits the detection of spatiotemporal characteristics of brain function by measuring the concentrations of oxygenated haemoglobin, [oxy-Hb], and deoxygenated haemoglobin, [deoxy-Hb], which are assumed to reflect cerebral blood flow (CBF). Since there are many types of NIRS devices, the results of the different investigations are given using: (1) solely [oxy-Hb], (2) [oxy-Hb] and [deoxy-Hb], (3) [oxy-Hb], [deoxy-Hb] and [total-Hb], or (4) using a tissue oxygenation (TOI)/saturation index/regional saturation index (rSO_2_) [21]. In a sense, they are all based on the same principle: an increase in CBF, an increase in [oxy-Hb] and a decrease in [deoxy-Hb] are all seen in active brain regions while people are participating in cognitive tasks; this principle is the basis of neuroimaging techniques such as NIRS. In our study, NIRS specifically provides a measure of rSO_2_ and is highly sensitive to conditions that alter the flow of oxygenated blood to brain tissue. There are indications that NIRS is sensitive enough to also detect small metabolic changes during the performance of cognitive tasks, including VFT by letters or categories [20] and they reveal that the VFT was the most widely used task in understanding impaired activation [19] or decreased cognitive performance. Performances, especially in the letter version of VFT, are considered to be mainly related to frontal lobe function, particularly within the left hemisphere. In studies using NIRS, the relationships between sleep deprivation and the reactivities of the cerebral cortex were evaluated as changes in [oxy-Hb] and [deoxy-Hb], respectively, during a VFT and concluded that decreased concentration and attention due to sleep deprivation can decrease task performance and was considered to be related to a decrease in PFC reactivity through a negative correlation with [oxy-Hb] changes and [oxy-Hb] increase [22].

It therefore seems reasonable to postulate that a decreased CBF reflects poor brain health, which in turn could result in poor cognitive performance. We speculate that this relationship could be explored using NIRS as a brain health indicator (CBF) or at least a lack of correct self-regulation of the PFC in the case of healthy individuals, given the lack of studies that relate this fact to night shift and sleep deprivation, as a fundamental contribution of our study. To our knowledge, no researchers have provided data to support this speculation.

Further to this, episodes of prolonged wakefulness impact negatively on mood. It has been suggested that sleep deprivation increases anxiety, irritability and depression scores [23,24]. The PFC plays an important role in emotion and is the region that is most sensitive to the effects of stress exposure [25]. Exposure to uncontrollable stress, which can lead to anxiety and/or burnout, causes impairment in PFC function reflected by rSO_2_ measures that can involve a decreased cognitive performance.

The purpose of this study was to investigate the effects of the night shift on cognitive performance and cerebral oxygenation/haemodynamics. In the present study, we used the NIRS system to measure baseline cerebral oxygenation levels (CBF by rSO_2_) in ICU nurses, we related their baseline oxygenation levels to night and day shift and to cognitive task performance and we evaluated the alterations in the brain function as PFC reactivity, assessed as changes in [oxy-Hb] and [deoxy-Hb], which reflect rSO_2_. We hypothesized that participants would experience fatigue, burnout, sleep deprivation and anxiety such that their performance in a VFT, measures of rSO_2_ and cerebral PFC reactivity after night duty would be impaired.

## 2. Materials and Methods

### 2.1. Study Population and Setting

We conducted a prospective, observational, comparative, randomized, cross-over study. Nurses and nursing assistants (n = 74) working in two ICUs in University Hospital, Badajoz, Spain, were prospectively included. All fulfilled the inclusion and exclusion criteria. Exclusion criteria were (1) a history of sleep disorder or serious comorbid illness; (2) current use of prescription sedative or stimulant; (3) not engaged in at least three night shifts during a 30-day period. All documents and measures were completed face-to-face within the ICU, with one of the study authors previously trained for it.

### 2.2. Design

Each nurse was their own control. Each subject participated in the study under two different conditions: once at the end of a night shift (NS) and once after a night of rest at home (NR). For each of the two groups, randomization using sealed envelopes was used to determine the order of the two conditions (NS first or NR first). These two evaluations were separated by a period of at least 14 days. The NR condition took place at least 3 days after the last on-duty shift. The duration of the night shift was 7 h (from 10:00 p.m. to 8:00 a.m.).

### 2.3. Instruments and Measures

#### 2.3.1. Clinical Interview

A clinical interview was used to assess self-reported sociodemographic data, lifestyle and substance use (coffee and tobacco), time worked in ICU, total time worked, use of relaxation techniques, average number of night shifts per month, number of declared hours of sleep during night shift, availability of sufficient time to do the job in one shift, intention to leave or change ICU, and future prospects of continuing in the unit.

#### 2.3.2. State-Trait Anxiety Inventory (STAI)

The State-Trait Anxiety Inventory [26] is a self-report 40-item measure to assess both the presence and severity of usual propensity to be anxious (Trait scale) and current symptoms of anxiety (State scale). Each item is rated on a 4-point Likert scale (1 to 4). The total score for both scales ranges from 20 to 80. Higher total scores represent higher anxiety severity.

STAI is a reliable and useful instrument to assess trait and state anxiety in a variety of contexts and populations. Convergent and discriminant validity, test–retest reliability and internal consistency of the Spanish adaptation of the instrument are good [27].

#### 2.3.3. Copenhagen Burnout Inventory (CBI)

The Copenhagen Burnout Inventory (CBI) is a 19-item self-report measure with three subscales [28]. Six items measure personal burnout (CBIpb), seven items relate to work-related burnout (CBIwb), and six items relate to client-related burnout (CBIcb). Twelve items use response categories according to frequency along a five-point Likert scale, of 100 (always), 75 (often), 50 (sometimes), 25 (seldom) and 0 (never/almost never). Seven items use response categories according to intensity, including “to a very high degree, a high degree, somewhat, a low degree, and very low degree”. Subscale scores of the CBI produce an index score from 0 to 100 where scores of 0–49 indicate “no burnout,” 50–74 are considered “moderate” and 75–99 “high”. A score of 100 is considered severe burnout. The CBI has been translated and used in a variety of countries, such as Spain [29], and it has been demonstrated that the Spanish version of the CBI is a reliable and valid instrument for measuring burnout.

#### 2.3.4. Verbal Fluency Task (VFT) and Procedure

A VFT was employed to test cognitive functions while assessing PFC haemodynamics by NIRS. Tests of verbal fluency (VF) were performed on each subject in the two conditions: NR and NS. The VF evaluation was divided into two tests: (1) First, a verbal semantic fluency test (VSF), where the subject is asked to name all the elements within a given semantic category (animals, plants and tools); (2) Second, a phonological verbal fluency test (FVF), in which the subject is asked to say all the words that begin with a particular syllable or letter (pa, la, ro, o, z). Each block lasts 60 s using a period of 20 s for each semantic category, syllable or given letter with a rest interval of 10 s every time a new one is introduced. In order to avoid the effect of memory, the semantic categories, syllables and letters were modified in each condition. In NR condition we used three categories (animals, plants and tools), two syllables (te, pa) and one letter (o). In the NS condition, subjects were asked to list food, sports and films categories and words that start with /la/, /ro/ and /z/. Incorrect responses included saying “pass”, listing peoples’ names, repeating words or producing grammatical variations of a previous word. Behavioural performance was assessed as the total number of correct words generated.

#### 2.3.5. NIRS Measurements and Procedure

The INVOS 5100 Cerebral Oximeter (Somanetics Corporation, Troy, MI, USA) was used to measure rSO_2_ in the dorsolateral PFC bilaterally. NIRS provides measures of [oxy-Hb] and [deoxy-Hb]. Near-infrared light absorption by [oxy-Hb] and [deoxy-Hb] was calculated using a modified Beer–Lambert Law. The relative amounts of both are used to calculate rSO_2_ and their cortical concentration changes are used as an indirect indicator of regional brain activation. The relationship between a decrease in [deoxy-Hb] (and consequently an increase in rSO_2_) and an increase in the blood-oxygenated-level-dependent signal of NIRS is a measure of cerebral activation. rSO_2_ was calculated assuming an arterial to venous blood ratio of 25:75%. The INVOS provides real-time measurement and a display of rSO_2_ in the microvasculature beneath the sensor. The two disposable LED sensors alternated between emitting 710 and 830 nm wavelengths of light, absorbed by haemoglobin. The two receiving optodes were 3 and 4 cm in distance from the LED. Light travels from the sensor’s light-emitting diode to either a proximal or distal detector, permitting separate data processing of shallow and deep optical signals. 

NIRS assessments have been demonstrated to provide a metric of cognitive activation similar to functional magnetic resonance imaging during cognitive performance tasks [30]. 

Before the beginning of the task, participants were instrumented with sensors for the left and right frontal lobes at the dorsolateral level of the PFC. The sensors were correctly secured in place.

rSO_2_ was measured while the participant performed a word fluency task under the following conditions: (1) rest (pre-test baseline, 1 min); (2) verbal fluency test (2 min); (3) rest (post-task baseline, 1 min) in NR and NS conditions. The measurements obtained will be named as follows: NR condition by rSO_2_-NR_1_, rSO_2_-NR_2_, rSO_2_-NR_3_, average rSO_2_-NR and NS condition by rSO_2_-NS_1_, rSO_2_-NS_2_, rSO_2_-NS_3_, average rSO_2_-NS. Throughout this period, the subject sat on a comfortable chair in a room that was illuminated by daylight. The sitting position is necessary to ensure comparability across studies because of spontaneous physiological oscillations, which could influence the NIRS signal quality and are posture dependent [31]. A mean was determined from the values recorded from two channels in the dorsolateral area of the PFC.

#### 2.3.6. Statistical Analysis

Data were analysed by IBM^®^SPSS^®^ Statistics 22. 

The variables were studied both from a descriptive and an inference point of view. The descriptive part was made in terms of mean ± standard deviation, percentages and correlation coefficients. The inference was carried out by a correlation test based on Pearson’s r coefficient and one-way ANOVA, followed by Tukey’s HSD multiple comparison method, *t*-paired test and Fisher’s exact test. A value *p* < 0.05 was considered significant. 

We also distinguish between two stages in the analysis depending on the moment (NR or NS condition). In the first stage, the generic variables and those measured during a day shift (after resting during the previous night) were analysed; in the second stage, the changes (worsening) measured during a posterior night shift were included in the analysis.

## 3. Results

The study began with 74 subjects. A short descriptive analysis of the generic variables measures gave the following results: the average age of the 74 subjects in this study was 45.82 ± 7.85 and 85% of them were women. They had been working in the ICU for an average of 11.05 ± 8.32 years (average of 17.41 ± 7.79 total years worked throughout their professional healthcare life). They worked on average 5.72 ± 1.03 nights a month on the night shift (Table 1).

Of the total number of subjects in the study, 66.2% said they stayed awake during night shifts, while 23% and 10.8% admitted that they slept for one and two hours, respectively; 71% had family members in their care; 29.7% practised sport regularly, 8.1% practised relaxation exercises, 13.5% practised both sport and relaxation exercises and 48.6% practised neither sport nor relaxation exercises; 27% of subjects were smokers; and 77% regularly drank coffee. Finally, 43.2% stated that they did not intend to stay in the ICU in the long term (Table 1).

### 3.1. Day Shift

The mean STAI score was 18.36 ± 9.78; the mean score in VFT was 44.59 ± 9.26. The average of the three rSO_2_ in NR condition (rSO_2_-NR_1_, rSO_2_-NR_2_, rSO_2_-NR_3_) was 65.78 ± 7.16 for the left side and 67.38 ± 8.14 for the right side (the evolution of oxygenated CBF is described below).

The scores in the three dimensions of burnout were the following: 39.95 ± 17.40 for CBIpb, 41.20 ± 15.05 for CBIwb and 31.24 ± 16.61 for CBIcb. The three dimensions of burnout were directly correlated. Nevertheless, they did not have a significant correlation with the rest of the variables except for the STAI-NR score. The correlation analysis showed a significant positive correlation between the STAI-NR score with the average values of the total burnout scores and their relative dimensions. Correlation coefficients were: r = 0.598 (*p* < 0.001) with CBIpb, r = 0.561 (*p* < 0.001) with CBIwb and r = 0.240 (*p* = 0.039) with CBIcb.

### 3.2. Night Shift

CBI dimensions and hours of sleep during the night shift indicated a significant positive correlation, which we also found with the STAI-NS score and with almost all the other variables (Table 2). In this case, when we studied this correlation, we applied one-way ANOVA followed by Tukey’s HSD post hoc method, since hours of sleep was considered in a categorical way, distinguishing only between 0, 1 or 2 h. In this sense, we found a significant positive correlation with all of the variables but CBIwb (Table 2). Nevertheless, this association could be summarized as follows: the group of subjects that slept 2 h differed significantly from 0 and 1 groups, but we did not find any significant differences between 0 and 1.

The group of eight subjects (Table 2) who said that they slept two hours during the night shift showed higher values on average for the STAI score, CBlpb and, extremely high for CBIcb. Interestingly, they showed the highest values on average in rSO_2_-NS for both sides while their scores in the VFT were clearly worse. Additionally, 87.5% (seven out of eight) said that they did not intend to stay in the ICU in the long term as compared to 37.9% for the rest of the values. This difference is significant (*p* = 0.018) according to Fisher’s exact test. These facts, in particular the poor result of the VFT, could be interesting for further study, but caused us to have serious doubts about the attitude of these subjects when performing the tests, especially the VFT. From a statistical point of view, a poor performance in VFT plus the longer resting time than the whole sample does not explain the variability and could jeopardize our conclusions, so we were forced to exclude these eight participants from the sample of the analysis.

At this stage of the analysis, only the remaining 66 subjects were considered. They showed a significant decrease in VFT on average, namely 8.53 ± 8.49 (*p* < 0.001). In general, there was also a significant increase in the STAI-NS score (3.17 ± 7.56, *p* = 0.001). Nevertheless, evolution in the STAI score depended on previous status: in fact, 19 subjects had a pre-pathological STAI-NR score (in the first test during the day shift), while the other 49 did not. The STAI-NS score increased significantly for the second group (5.70 ± 6.91, *p* < 0.001)) although it decreased significantly in the first (3.10 ± 5.08, *p* = 0.016).

The evolution of the CBF (rSO_2_ measures) on the left side and on the right side, from the first measure in the day shift to the last at the end of the night shift, is shown in Table 3. For each side, the Student’s *t*-paired test and Pearson’s correlation test were applied to analyse changes in the mean with respect to the prior measure, as well as the correlation with it. 

**Figure 1:**
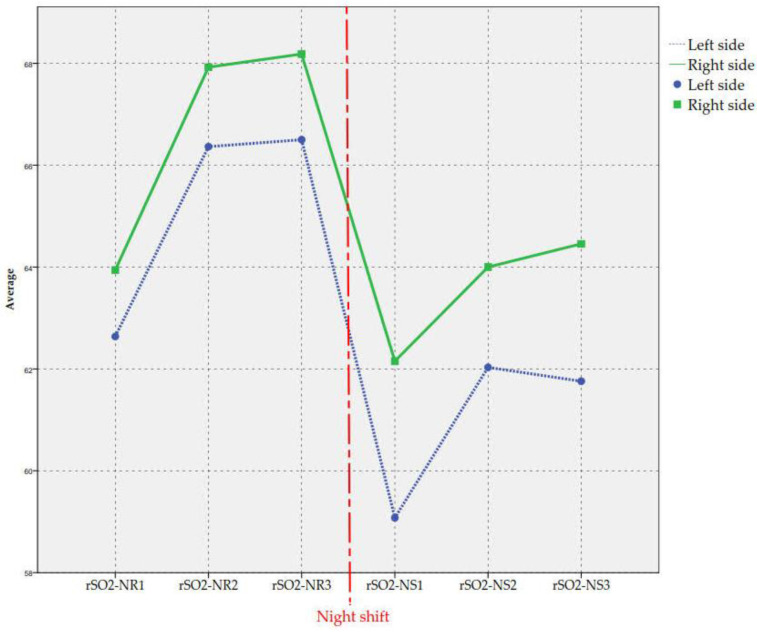
Evolution of average rSO_2_ scores.

Differences between the means on the left and the right side at the same time, as well as the correlation between both, were analysed in the same way, as shown in the right columns of the table (Table 3). Finally, the last two rows show the same analysis for the means of the three measures in NR and NS conditions. Figure 1 illustrates this evolution. The vertical line expresses the discontinuity between the third measure of the day shift and the first of the night shift, since there are several days between both.

As summarised in Table 3 and Figure 1, average rSO_2_ decreased significantly in the night shift in both sides. Correlations between both sides are stronger during the day shift. The second measures were clearly larger than the first in both sides and in both shifts while the third measures were similar to the second ones. rSO_2_ was better on average for the right side at every stage. These differences turned out to be significant except for rSO_2_-NS_2_.

In a second stage of analysis, we tried to explain the link between the STAI score, VFT changes (worsening) and average rSO_2_ during the night shift. Before, we highlighted that the STAI score in the NR condition showed an inverse and significant correlation with VFT in the same condition (r = −0.267, *p* = 0.030). In other words, at the beginning, the higher the STAI score, the worse the VFT. Nevertheless, we already know that VFT became worse on average during the night shift (8.53 ± 8.49, *p* < 0.001) but this worsening was lower for those whose previous STAI score was greater, since the correlation between the STAI-NR and the worsening in VFT was r = −0.248 (*p* = 0.045).

**Figure 2:**
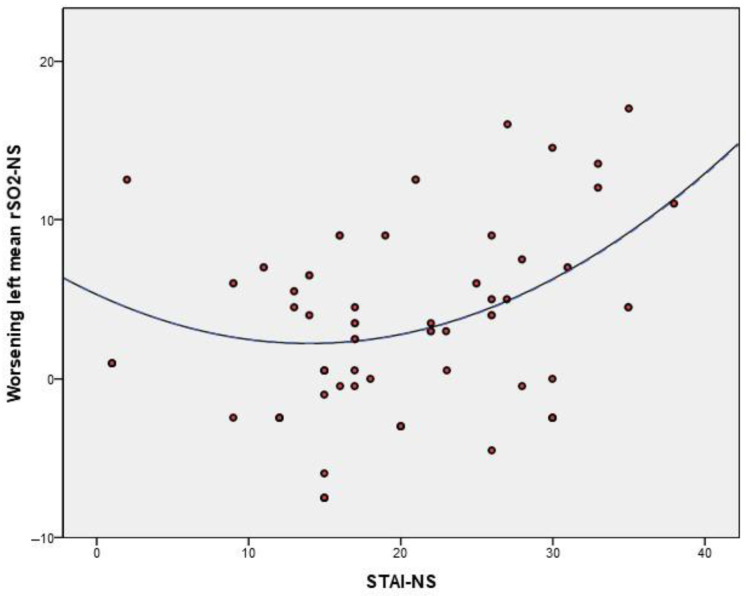
Relationship between the STAI score in NS condition and decrease rSO_2_ in the left side.

The decrease in average rSO_2_ in the left side during the night shift correlated directly with STAI in the NR (r = 0.253, *p* = 0.041) and NS conditions (r = 0.325, *p* = 0.008), i.e., the greater the decrease on average rSO_2_ from the NR condition to the NS, the higher the STAI score during the night shift. However, this statement should be qualified, since in this latter case we also observed a moderate decrease in the left average rSO_2_-NS for the subjects with the lowest STAI score in the NS condition. A quadratic adjustment (Figure 2) gave a correlation coefficient of R = 0.450. Therefore, we can associate median STAI scores in the NS condition with the lowest worsening in the left average rSO_2_-NS.

## 4. Discussion

As far as we know, the present study is the first to attempt to evaluate the disturbances in the brain functions of on-duty nurses by direct assessments of cerebral haemodynamic reactivity measured by PFC oxygenation (decreased rSO_2_) using a non-invasive NIRS method. Although similar studies have been conducted, they have focused on on-duty medical residents [32,33]. It was found that cerebral perfusion may be an important mechanism, as blood flow, especially to the PFC, directly governs oxygen delivery [34]. Through analysis of the NIRS data, our results suggest that sleep deprivation is related to the decreased reactivity of the PFC. We found that the night shift affected brain activity, having confirmed a decrease in rSO_2_ in the prefrontal region after the night shift, likely mediated by the sleep deprivation and fatigue induced by this shift, with respect to the scores found in the day shift. This is in line with previous studies linking sleep deprivation and PFC reactivity exploring changes in [oxy-Hb]. A negative correlation was found between these factors and the decrease in [oxy-Hb] [22].

To this we add that the inverse correlation found in our study between VFT score and night shift is also likely related directly to decreased sleep. Scott et al. (2010) conducted a study with 2624 registered nurses (RNs) to investigate potential reasons why they may obtain less sleep. The findings of the study revealed that nurses on night shift had greater odds of sleep deprivation because of rapid returns to work and working the opposite of morningness or eveningness compared to their dayshift counterparts [35]. Morningness and eveningness refer to the individual differences in circadian rhythm and preference for early or late awakening and cortisol levels [36]. Sleep deprivation affects the functioning of certain brain areas and thus impairs cognitive performance. Cognitive impairments in nurses during the night shift as opposed to the day shift may be mediated predominantly through the decreased alertness and attention that occurs with wake-state instability. This is consistent with previous research, although not focused on nurses [11,12,33].

Furthermore, we focused on cerebral oxygen availability via CBF, as indexed from measures of rSO_2_. Availability of oxygen is crucial for cognitive processes to be intact and a lack of oxygen in the brain leads to lower cognitive performance [37]. Brain activity leads to increased oxygen consumption, which is accompanied by an increase in CBF due to neurovascular coupling [38]. Research indicates that it is advantageous to have a higher cerebral oxygenation level, owing to the increased oxygen availability facilitating cognition [39], and NIRS can measure the small changes in blood oxygenation induced by cognitive processing. In the present study, when exploring the relationship between VFT, night shift and CBF, we found a significant decrease in VFT and rSO_2_ during the night shift compared to the day shift. Cognitively, when a VFT is enunciated, it creates a meta-objective and activates in the person a word-search mechanism. This search involves an effort, which can be considered of an executive type, since it keeps the target signal set by the task, and this depends on different cognitive capacities that intervene in VFT performance, the main ones being related to the prefrontal and temporal activity of brain [40]. Worsening in cognitive performance, therefore, at least in part, may be associated with the sleep-deprivation-induced decrease observed in the night shift in the brain oxygen supply. Moser et al. (2012) showed that PET measures of baseline CBF were related to general cognitive functioning, even when controlling for age and sex and without evidence of neurological disease. This study suggests that cerebral health may be related to baseline CBF, which is also related to cognitive functioning [41]. The relationship that we found, though with a different method, had already been verified by Suda et al., in their study of decreased cortical reactivity in 2008, concluding, as is our case, that the performance of this task was negatively correlated with the [oxy-Hb] changes during cognitive activation in a sleep deprivation situation [22]. In addition, Nishida et al. (2017), found that measures of fatigue and daytime sleepiness were significantly correlated with decreased cerebral activity in the resident physician after night duty [33]. Findings from functional brain imaging studies suggest reduced activity in PFC and one strong possibility: that the performance deficits that characterize sleep deprivation result specifically from the (de)activation of PFC [18]. In this sense, our results possibly indicate that sleep loss results in deactivation of the PFC regions, and such deactivation may underlie deficits in specific aspects of cognitive performance, such as performance in a VFT.

In this study, we also examined psychological stress and anxiety related to the night shift experience, in addition to that probably associated with the conditions of sleep deprivation that this shift entails. In general, there was also a significant increase in the level of anxiety due to the night shift compared to the day shift, coinciding with other previous studies that have also determined this association, although in physicians or medical residents [14,33,42]. It has been suggested that sleep deprivation increases anxiety, irritability and depression scores [23]. Therefore, we speculate that nurses suffer considerable psychological stress during the night shift. Nurses working night shifts and rotating shifts struggle more to stay awake during their work activities compared to nurses working day/evening shifts. In accordance with these results, Ulas et al. (2012), showed a relationship between the effect of day and night shifts on the oxidative stress and anxiety symptoms of the nurses [43]. 

Our results also demonstrate a negative correlation between the STAI scores and the VFT, with worse VF results in nurses with higher anxiety scores, mainly on the night shift. These results are in disagreement with previous studies where no relationship was found between STAI and VFT [22]. Our findings suggest that positive mood has a beneficial effect on VFT performance. Consequently, in the present study, alterations in mood may explain the observed detriments in task performance.

In addition, we found an inverse correlation between anxiety levels and PFC activity during the night shift: a higher level of anxiety results in a decrease in rSO_2_. This negative correlation is in disagreement with the results from other studies in which [oxy-Hb] changes did not correlate with STAI scores [22]. An increase in [oxy-Hb] and PFC activation is shown [44], but in agreement with the results of some studies, PFC has also been demonstrated to be critically involved in the mechanism underlying anxiety [45,46,47]. At the same time, these brain regions are involved in various cognitive processes influenced by anxiety and psychological stress. Previous data have reported that highly anxious individuals showed reduced PFC activity and a slower response to processing competition when the task did not fully occupy attentional resources [48]. This could explain the deficit and daily difficulties in cognitive performance tasks that are associated with clinical anxiety. Different paradigms showed points in common in a reduced response in the lateral prefrontal regions in highly anxious individuals. The PFC is involved in the management of task processing and the altered functioning of this circuit is suggested in high-anxiety states [45,46,48].

In summary, our results show a significant decrease in VF, an increase in the level of anxiety and a decrease in CBF by rSO_2_ measures during the night shift, mainly in the left side. In this sense, cumulative neuropsychological evidence suggests that anxiety has an impact on prefrontal cognitive processes; these processes could be biased by individual differences in anxiety-related characteristics under psychological stress [49]. Anxiety is associated with performance impairment in numerous cognitive tasks and highly anxious individuals show reduced prefrontal activity [48].

Finally, in our attempt to relate sleep deprivation, CBF (rSO_2_) in PFC and possible modifications of VFT with burnout in ICU nurses, we did not find any significant statistical association between them. In contrast, surprisingly, a significant positive correlation was found between the mean scores of the dimensions of burnout (CBIpb, CBIwb and CBIcb) and total burnout with the number of hours of sleep they admitted during the night shift. This fact has also been pointed out by Giorgi et al. (2018). In the conceptual framework of their study, they proposed that there was a circular relationship between burnout and sleep loss, mediated by the effects of personal burnout on poor and impaired sleep, but they concluded from their results that in their model, sleep deprivation and burnout are affected by other factors that are outside of this circular relationship, such as gender or work setting [50].

In our case, we were able to link nurse burnout and anxiety. STAI scores positively correlated with burnout in all three dimensions. In this sense, we found no studies investigating this relationship using the same instrument for measuring burnout (CBI) with the anxiety levels of nurses, although there are on other healthcare professionals (midwives). With regard to our results, Fenwick et al. (2018) [10] reported that in a non-continuity care group, which would include our entire sample of ICU nurses working on a rotating shift, they not only suffered significantly higher levels of anxiety, but also higher rates of burnout in all three dimensions. This could explain the score levels found in our sample in the NR and NS conditions, both in CBI and STAI.

Our results support the growing body of evidence from different fields which demonstrates that care models based on shift work patterns cause high levels of fatigue, emotional distress and anxiety [51,52].

There are several limitations of this study. NIRS is limited to cortical layers because the penetration depth is, in general, less than half of the source-detector separation and it suffers from its vulnerability to changes in scalp blood flow and to changes in systemic physiology (e.g., increase in heart rate). Variations in the thickness of the skull and adjacent tissues can affect the inter-subject sensitivity of NIRS, especially in adults. Of note, while NIRS has proven to be a useful and reliable tool in some research fields, currently no standardized procedures regarding the processing of NIRS data are available. Moreover, the methods used to measure cortical haemodynamics during cognitive tasks are diverse. Brain function was monitored as “reactivity”, that is, the activity change from the baseline level, not as baseline activity, owing to the measurement nature of NIRS. Hence, the results obtained should be interpreted as showing the relationship between the desynchronization of circadian rhythm and brain function reactivity, not activity. Only the VFT was employed for cognitive activation. Different results can be obtained using other cognitive tasks, such as the continuous performance test, which enables the monitoring of sustained attention.

Given that mood may be associated with activation in deep brain areas, alterations in cerebral haemodynamics and oxygenation reflecting the observed altered mood states may not have been detected in this study. We assessed mood via the questionnaire only, and thus this should not be considered as an objective measure.

## 5. Conclusions

Sleep deprivation during the night shift was considered to be related to decreased dorsolateral PFC reactivity. After the night shift, the nurses showed a decrease in PFC activity and in cognitive performance. This result implies that the brain activity of the nurses exposed to stress, sleep deprivation and fatigue during the night shift was affected differently to that in the day shift. The degree of sleep loss could be associated with neurocognitive dysfunction and might have occupational and patient safety consequences. We strongly advise reconsideration of the shift assignments of and to seriously consider the implementation of continuity in nursing care models.

The present findings should be replicated in future research. With regard to assessment, given the relatively low cost and non-invasive nature of NIRS as an imaging tool, and findings suggesting it as a potential measure of cerebral health, it could be used to track the effects of sleep deprivation of nurses on shift work as a factor that is strongly associated with a profound desynchronization of circadian rhythm, and in particular with night-shift work, since it disrupts normal circadian physiology, as well as tracking changes in both brain functioning and cognitive performance.

## Figures and Tables

**Table 1:** Sociodemographic data and characteristics of the sample.

Variable	Categories	N (%)	M ± SD
Age (years)	Males Females Total	11 (14.9) 63 (85.1) 74 (100)	38.3 ± 6.0 47.1 ± 7.40 45.8 ± 7.84
Marital status	Married Single Divorced/Widowed	43 (58.1) 21 (28.4) 10 (13.5)	
Childcare or elderly	Yes No	53 (71.6) 21 (28.4)	
Relaxation	Yes No	38 (51.4) 36 (48.6)	
Type	Sport Relaxation techniques More than one None	22 (29.7) 6 (8.1) 10 (13.5) 36 (48.6)	
Smokes	Yes No	20 (27.0) 54 (73.0)	
Coffee	Yes No	57 (77.0) 17 (23.0)	
Management	Yes No	25 (33.8) 49 (66.2)	
Time worked in ICU			11.1 ± 8.3
Total time worked			17.4 ± 7.8
Availability of enough time to perform job in one shift	Yes No	47 (63.5) 27 (36.5)	
Future prospect of continuing in the unit	Yes No	42 (56.8) 32 (43.2)	
Intention to leave or change ICU	Yes No	30 (40.5) 44 (59.5)	
Number of hours of sleep during night shift	0 1 2	49 (66.2) 17 (23.0) 8 (10.8)	
Number of night shifts per month	4 5 6 7 8 9	10 (13.5) 14 (18.9) 43 (58.1) 3 (4.1) 2 (2.7) 2 (2.7)	

Abbreviations: M, mean; SD, standard deviation.

**Table 2:** Relationship between number of hours of sleep and the main variables measured in NS condition.

NS Condition	Nº Hours Asleep during Night Shift			
	0 (N = 49)	1 (N = 17)	2 (N = 8)	
	M ± S.D.	M ± S.D.	M ± S.D. *	ANOVA
STAI score	17.51 _a_ * ± 9.19	15.82 _a_ ± 10.01	29.00 _b_ ± 6.23	*p* = 0.030
VFT	45.96 _a_ ± 9.00	44.47 _a_ ± 10.03	36.50 _b_ ± 4.72	*p* = 0.025
Average rSO_2_-NS Left side	65.87 _a_ ± 8.01	63.13 _a_ ± 3.19	70.87 _b_ ± 4.84	*p* = 0.039
Average rSO_2_-NS Right side	67.58 _a_ ± 9.16	64.10 _a_ ± 3.65	73.17 _b_ ± 4.40	*p* = 0.030
CBlpb	36.63 _a_ ± 15.00	42.11 _a,b_ ± 21.34	55.70 _b_ ± 14.25	*p* = 0.012
CBIwb	39.49 _a_ ± 14.94	43.06 _a_ ± 13.90	47.75 _a_ ± 17.71	*p* = 0.304
CBIcb	28.13 _a_ ± 15.30	31.35 _a_ ± 16.92	50.00 _b_ ± 12.20	*p* = 0.002

Abbreviations: M, mean; SD, standard deviation. * _a,b_ in table indicate that there is a significant difference between the two groups according to Tukey’s method only if they do not have any letters in common.

**Table 3:** Evolution of rSO_2_ measures.

Measures (N = 66)	Left Side		Right Side		Both Sides	
	M ± S.D. ^a^	Correlation ^b^	M ± S.D. ^a^	Correlation ^b^	R-L: M ± S.D. ^c^	Correlation ^d^
rSO_2_-NR_1_	62.64 ± 7.18	-	63.94 ± 8.02	-	1.30 ± 4.95, *p* = 0.033	r = 0.802, *p* < 0.001
rSO_2_-NR_2_	66.36 ± 7.25, *p* < 0.001	r = 0.928, *p* < 0.001	67.92 ± 8.32, *p* < 0.001	r = 0.928, *p* < 0.001	1.56 ± 4.42, *p* = 0.006	r = 0.847, *p* < 0.001
rSO_2_-NR_3_	66.50 ± 7.88, *p* = 0.725	r = 0.918, *p* < 0.001	68.18 ± 9.18, *p* = 0.547	r = 0.894, *p* < 0.001	1.68 ± 4.84, *p* = 0.006	r = 0.849, *p* < 0.001
rSO_2_-NS_1_	59.08 ± 7.96, *p* < 0.001	r = 0.603, *p* < 0.001	62.15 ± 9.04, *p* < 0.001	r = 0.693, *p* < 0.001	3.08 ± 8.01, *p* = 0.003	r = 0.553, *p* < 0.001
rSO_2_-NS_2_	62.03 ± 7.97, *p* < 0.001	r = 0.929, *p* < 0.001	64.00 ± 9.28, *p* < 0.001	r = 0.959, *p* < 0.001	1.97 ± 8.21, *p* < 0.056	r = 0.555, *p* < 0.001
rSO_2_-NS_3_	61.76 ± 8.77, *p* = 0.441	r = 0.946, *p* < 0.001	64.45 ± 9.86, *p* = 0.196	r = 0.958, *p* < 0.001	2.70 ± 8.97, *p* = 0.017	r = 0.542, *p* < 0.001
M rSO_2_-NR	65.17 ± 7.17	-	66.68 ± 8.22	-	1.52 ± 4.21, *p* = 0.005	r = 0.859, *p* < 0.001
M rSO_2_-NS	60.95 ± 8.01, *p* < 0.001	r = 0.725, *p* < 0.001	63.54 ± 9.24, *p* = 0.001	r = 0.673, *p* < 0.001	2.58 ± 8.16, *p* = 0.013	r = 0.560, *p* < 0.001

Abbreviations: M, mean; SD, standard deviation. ^a^ *p*-value corresponds to mean comparison with previous stage in the same side according to *t*-paired test. ^b^ *p*-value corresponds to correlation with previous stage in the same side according to Pearson’s correlation test. ^c^ *p*-value corresponds to mean comparison between both sides (right–left) at the same stage according to *t*-paired test. ^d^ *p*-value corresponds to correlation between both sides at the same stage according to Pearson’s correlation test.

## Data Availability

The data underlying this article cannot be shared publicly to maintain the privacy of the individuals that participated in the study. The data will be shared upon reasonable request to the corresponding author.
